# Dysregulation of Neuronal Iron Homeostasis as an Alternative Unifying Effect of Mutations Causing Familial Alzheimer’s Disease

**DOI:** 10.3389/fnins.2018.00533

**Published:** 2018-08-13

**Authors:** Amanda L. Lumsden, Jack T. Rogers, Shohreh Majd, Morgan Newman, Greg T. Sutherland, Giuseppe Verdile, Michael Lardelli

**Affiliations:** ^1^College of Medicine and Public Health, Flinders University, Adelaide, SA, Australia; ^2^South Australian Health and Medical Research Institute, Adelaide, SA, Australia; ^3^Neurochemistry Laboratory, Department of Psychiatry-Neuroscience, Massachusetts General Hospital (East), Harvard Medical School, Harvard University, Charlestown, MA, United States; ^4^Neuronal Injury and Repair Laboratory, Centre for Neuroscience, College of Medicine and Public Health, Flinders University, Adelaide, SA, Australia; ^5^Centre for Molecular Pathology, School of Biological Sciences, University of Adelaide, Adelaide, SA, Australia; ^6^Discipline of Pathology, Sydney Medical School, University of Sydney, Sydney, NSW, Australia; ^7^School of Pharmacy and Biomedical Sciences, Faculty of Health Sciences, Curtin Health Innovation Research Institute, Curtin University, Bentley, WA, Australia

**Keywords:** iron homeostasis, trafficking, familial Alzheimer’s disease, neurodegeneration, mutation, secretase, PRESENILIN, AMYLOID BETA A4 PRESCURSOR PROTEIN

## Abstract

The overwhelming majority of dominant mutations causing early onset familial Alzheimer’s disease (EOfAD) occur in only three genes, *PSEN1, PSEN2*, and *APP*. An effect-in-common of these mutations is alteration of production of the APP-derived peptide, amyloid β (Aβ). It is this key fact that underlies the authority of the Amyloid Hypothesis that has informed Alzheimer’s disease research for over two decades. Any challenge to this authority must offer an alternative explanation for the relationship between the *PSEN* genes and *APP*. In this paper, we explore one possible alternative relationship – the dysregulation of cellular iron homeostasis as a common effect of EOfAD mutations in these genes. This idea is attractive since it provides clear connections between EOfAD mutations and major characteristics of Alzheimer’s disease such as dysfunctional mitochondria, vascular risk factors/hypoxia, energy metabolism, and inflammation. We combine our ideas with observations by others to describe a “Stress Threshold Change of State” model of Alzheimer’s disease that may begin to explain the existence of both EOfAD and late onset sporadic (LOsAD) forms of the disease. Directing research to investigate the role of dysregulation of iron homeostasis in EOfAD may be a profitable way forward in our struggle to understand this form of dementia.

## Iron, Inflammation, and Neurodegeneration

Iron is the most abundant element in our planet and the most abundant transition metal in the human body (Emsley, 1998). It has been a central component of energy metabolism since the dawn of life, over one billion years before oxygenation of the Earth’s atmosphere drove the evolution of energy production by oxidative phosphorylation (Sheftel et al., 2012). Iron has been used medicinally for thousands of years before elucidation of its role in biology began with its discovery as a component of our blood [announced in 1746 (Busacchi, 1958; Sheftel et al., 2012)].

The most reactive form of iron, ferrous iron, Fe^2+^, was abundant in the biosphere before oxygen formation by cyanobacteria depleted it to form less soluble ferric (Fe^3+^) oxide. It is Fe^2+^ that is required to form the iron–sulfur complexes that are essential for aerobic respiration by bacteria and, consequently, for the function of mitochondria in our eukaryotic cells. However, the exploitation of Fe^2+^ in redox reactions involves the unavoidable generation of oxidative and nitrosative stress that must be properly managed lest it damage mitochondria, damage their antioxidant controls, and generate additional stress in a positive feedback loop (Leandro et al., 2015). Levels of bioavailable iron are tightly regulated by balancing of iron storage and iron acquisition through mechanisms that are exquisitely sensitive to iron requirements. Iron is normally acquired from the diet by absorption across enterocytes, transported around the body chaperoned by TRANSFERRIN, internalized by iron-requiring cells via TRANSFERRIN RECEPTOR (TFRC)-mediated endocytosis [and other pathways (Mills et al., 2010; Prus and Fibach, 2011)], and released from the endolysosomal compartment into the cytosol for cellular use. Excess iron is stored in numerous ways: in the cytosolic iron storage protein complex, FERRITIN; within mitochondria in MITOCHONDRIAL FERRITIN (FTMT, Levi et al., 2001); and in protein/chemical complexes such as hemosiderin (Iancu, 2011) and neuromelanin (Double et al., 2003) apparently in the lysosomal pathway (Iancu, 2011; Plum et al., 2016). Some cell types such as enterocytes, macrophages and hepatocytes have evolved increased iron storage capacity (Linder, 2013). Iron is also exported from cells via the protein SOLUTE CARRIER FAMILY 40 (IRON-REGULATED TRANSPORTER), MEMBER 1 (SLC40A1, better known as FERROPORTIN 1 or FPN1) (Donovan et al., 2000). FPN1 acts in conjunction with an iron oxidase (to convert Fe^2+^ to Fe^3+^) such as HEPHAESTIN (in intestinal epithelial cells absorbing iron in the gut) (Vulpe et al., 1999) or the homologous protein CERULOPLASMIN (in most other cell types) (Takahashi et al., 1984; Wang and Wang, 2018). The intracellular trafficking of iron within neurons will be discussed in more detail later.

Iron, in particular Fe^2+^, is so important for bacterial growth that denying its availability to bacteria is central to our innate immune defense against infection. In response to infection, an antibacterial peptide hormone, HEPCIDIN ANTIMICROBIAL PEPTIDE (HAMP), is produced (Pigeon et al., 2001). HAMP signaling drives internalization and degradation of FPN1 (Wang S.M. et al., 2010; Wessling-Resnick, 2010). This prevents dietary iron from exiting enterocytes into the circulation, and stored iron from exiting macrophages and other cells that have evolved to store it. This underlies the anemia associated with acute and chronic disease (Roy and Andrews, 2005). The iron storage capacity in mammalian systems is increased by upregulation of FERRITIN (Konijn and Hershko, 1977; Rogers, 1996), and FTMT (Yang et al., 2015). Another important protein that is a part of the mammalian innate immune system is the iron-binding protein LACTOTRANSFERRIN (LTF) that shows structural homology to TRANSFERRIN and has anti-microbial and anti-inflammatory activities (reviewed by Bonaccorsi di Patti et al., 2018). Of course, in defending against bacteria, the sequestration of iron also affects the functionality of our own mitochondria so that, during inflammation, cells may rely more heavily on energy generation by glycolysis (Gaber et al., 2017).

Inflammation is recognized as a key, unifying characteristic of all forms of neurodegeneration (Richards et al., 2016) so it is unsurprising that iron appears to play key roles in many neurodegenerative diseases. Some examples of this are to be provided but the main aim of this paper is to examine the role iron may play in the most common form of dementia, Alzheimer’s disease.

## Alzheimer’s Disease

The current criteria for the pathological diagnosis of Alzheimer’s disease are used to compose an “ABC” score. The “A” describes the extent of Aβ peptide immunostaining (Thal et al., 2002) while “B” is derived from argyrophilic neurofibrillary tangles (NFTs, composed largely of hyperphosphorylated MICROTUBULE-ASSOCIATED PROTEIN TAU, MAPT) (Braak and Braak, 1991) and “C” from argyrophilic neuritic plaques (Mirra et al., 1991). Intermediate or high ABC scores are regarded as consistent with Alzheimer’s disease (Montine et al., 2012). Aβ is first seen throughout the ventral neocortex before spreading to the medial temporal lobe structures. In contrast, NFTs are first observed in the transentorhinal cortex before spreading throughout the hippocampal formation and neocortex with relative sparing of primary cortices (Braak and Braak, 1991). Unlike the linear course of events of Aβ aggregation followed by effects on MAPT that is described by the “Amyloid Hypothesis” (Hardy and Selkoe, 2002) there tends not to be a temporal or physical overlap of NFTs and Aβ in the cortex (Nelson et al., 2009). Indeed, it is the spread of NFTs rather than of Aβ plaques that is correlated with the severity and duration of Alzheimer’s disease (Arriagada et al., 1992). However, most neuritic plaques and some diffuse plaques are tau positive (Dickson and Vickers, 2001) while neuropil threads (composed of MAPT) are commonly seen among plaques suggesting that the overlap of Aβ and MAPT pathologies is more extensive that commonly appreciated when all types of MAPT pathology are considered.

Past and more recent research suggests that changes in iron trafficking and accumulation may be important in LOsAD. Two decades ago, Drs. Mark Smith and George Perry showed iron accumulation associated with the histological hallmarks of Alzheimer’s disease; Aβ plaques and NFTs (Smith et al., 1997) while Kennard et al. (1996) found that serum levels of the iron binding protein MELANOMA-ASSOCIATED ANTIGEN p97 (MFI2) were distinctly higher in individuals with Alzheimer’s disease compared to cognitively normal controls. More recently, the degree of iron accumulation in the brain has been seen to correlate with the degree of plaque and NFT pathology (van Duijn et al., 2017). While intracellular aggregation of Aβ may well contribute to plaque formation (Friedrich et al., 2010) and the rate of formation of an individual plaque is debated (Burgold et al., 2011; Hefendehl et al., 2011), the likelihood that microhemorrhages contribute to plaque formation is supported by their heme and fibrinogen content and their spatial correlation with blood vessels (Cullen et al., 2005, 2006). Since heme is rich in iron, an increasing brain Aβ plaque load would be expected to increase overall brain iron content.

Analysis of the iron storage complex FERRITIN shows great diagnostic and predictive potential for Alzheimer’s disease. In particular, FERRITIN levels in cerebrospinal fluid (an indicator of iron storage capacity) are very significantly higher in carriers of the major LOsAD risk allele, the 𝜀4 allele of the gene APOE (APOE4), and predict conversion from mild cognitive impairment (MCI) to Alzheimer’s disease (Ayton et al., 2015). Also, levels of FERRITIN in plasma correlate strongly with Aβ levels in the neocortex (as assessed by positron emission tomography) so that analyzing plasma FERRITIN may assist in identifying people at high risk for Alzheimer’s disease (Goozee et al., 2017).

## Uncertainty Regarding the Mechanistic Role of Aβ in Alzheimer’s Disease

As of June 2018, a search in PubMed for the term “Alzheimer’s disease” finds over 105,000 papers (excluding reviews). Nevertheless, there is still no consensus on the pathological mechanism underlying this disease. Indeed, the very definition of the disease itself is disputed (Herrup, 2015; De La Torre, 2016; Iturria-Medina et al., 2016; Whitehouse and George, 2016).

The past two decades have seen great enthusiasm for the Amyloid Hypothesis of Alzheimer’s disease that posits a pathological role for the Aβ peptide cleaved from the AMYLOID BETA A4 PRECURSOR PROTEIN (APP). The Amyloid Hypothesis is an irrefutable tenet for many in the Alzheimer’s disease research community since changes in Aβ production appear to be the only common mechanism linking the few loci where mutations causing early onset familial Alzheimer’s disease (EOfAD) are found. However, the mode of Aβ’s purported toxicity remains to be clearly defined and recent prominent review papers have admitted to serious incongruities between observations of Aβ deposition in the brain and the progress of the disease (Herrup, 2015; De Strooper and Karran, 2016). It is thus uncertain whether Aβ, accumulation of which is currently a required element in the definition of Alzheimer’s disease (McKhann et al., 2011) is a causative agent, a protective mechanism, or an ‘innocent bystander’ in the pathological process. [It is also worth reflecting on the fact that a naïve, most parsimonious interpretation of the presence of iron in the plaques and NFTs of Alzheimer’s disease brains (Smith et al., 1997) – that resembles so clearly the silver staining used by Alois Alzheimer himself to identify these histological landmarks (Alzheimer et al., 1995) – is that iron is the common causative agent rather than the Aβ peptide somehow driving both plaque and NFT formation and then these coincidentally both binding iron.]

**FIGURE 1:**
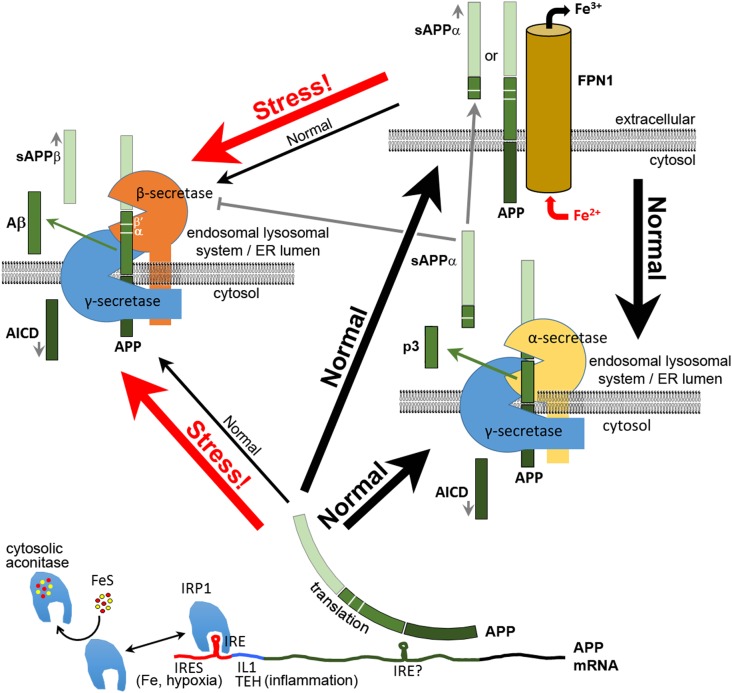
Multiple functions of APP in neurons. After its translation, APP can function in neurons to bind FPN1 and enhance the export of excess Fe^2+^. Some APP is cleaved by α-secretase or (mainly under stress) by β-secretase complexes, the latter producing the antimicrobial and antioxidant peptide Aβ. The secreted extracellular domains cleaved from APP by α–secretase (sAPPα) or β-secretase (sAPPβ) adopt different structures and activities. Like APP, sAPPα is able to increase the expression of FPN1 at the cell surface. sAPPα can also inhibit the activity of β-secretase. Translation of APP mRNA is suppressed by binding of IRP1 to an iron responsive element (IRE) (Rogers et al., 2002) when synthesis of Fe–S clusters is deficient. Under inflammatory stress and/or hypoxia, APP transcription is upregulated by oxidative stress, and APP translation is stimulated by binding of IL1 at a translation enhancer site (TEH) (Rogers et al., 1999) and by an internal ribosome entry site (IRES, Beaudoin et al., 2008) that permits translation in defiance of the unfolded protein response. The increased APP protein either increases iron export for sequestration by other cells or is diverted intracellularly to cleavage for production of Aβ and other APP proteolytic products. Plasma membrane APP may also be internalized for cleavage, probably due to interactions with SORTILIN and NGFR (p75) (Yang et al., 2013; Saadipour et al., 2017). β′ and α cleavage sites are indicated. It is not known what domains of α- and β-secretase interact with γ-secretase. Indeed, the functional existence of such complexes appears inconsistent with other data on the subcellular localization of these enzyme activities (e.g., see Pera et al., 2017). Note that the BACE1 protein that provides β-secretase activity was recently shown to be involved in intracellular Cu^2+^ homeostasis (Liebsch et al., 2017).

The dominant mutations causing EOfAD are now recognized to occur in four genes, *APP, PRESENILIN 1* (*PSEN1*), *PRESENILIN 2* (*PSEN2*), and (more recently) *SORTILIN-RELATED RECEPTOR 1* (*SORL1*). Readers can refer to a number of excellent reviews and research papers describing these mutations (Tanzi, 2012; Guerreiro and Hardy, 2014; Verheijen et al., 2016). Mutations in *SORL1* are thought to influence the production and secretion of Aβ by mechanisms that are not yet clearly defined (reviewed by, Yin et al., 2015). However, the involvement of *APP* and the *PSEN*s in production of Aβ is considered clear: the PSENs form the catalytic core of γ-secretase complexes that cleave a transmembrane fragment of APP to release Aβ (**Figure 1**). [Intriguingly, SORL1 is also a substrate of γ-secretase (Bohm et al., 2006)]. EOfAD mutations in APP are thought to affect either the production rate and/or form of Aβ while EOfAD mutations in the PSENs have been thought to enhance the production of the longer forms of Aβ, in particular the 42 amino acid residue form, Aβ_42_, relative to the predominant, 40 amino acid residue form, Aβ_40_ (Chavez-Gutierrez et al., 2012; Szaruga et al., 2015). However, this idea remains controversial since many of these mutations may decrease total γ-secretase activity and reduce overall Aβ production (Jayne et al., 2016; Sun et al., 2017), casting doubt on a causative role for Aβ in Alzheimer’s disease pathology.

The PSENs are polyfunctional proteins (reviewed by, Duggan and McCarthy, 2016; Jayne et al., 2016; Otto et al., 2016). When cleaved within their “cytosolic loop” domain they apparently become catalytically active in γ-secretase complexes. However, before cleavage, PSEN holoproteins are involved in other cellular activities. The best defined of these is the role of PSEN1 as a chaperone for presentation of the protein ATPase, H^+^ TRANSPORTING, LYSOSOMAL, V0 SUBUNIT A1 (ATP6V0A1) for *N*-glycosylation by the Sec61alpha/oligosaccharyltransferase complex (Lee J.H. et al., 2010; Lee et al., 2015). Correct *N*-glycosylation of ATP6V0A1 is required for its targeting to lysosomes (Lee J.H. et al., 2010) where it functions as a subunit of the vacuolar ATPase (v-ATPase) complex required for lysosomal acidification. Thus, EOfAD mutations in *PSEN1* appear to decrease the acidity, and reduce the activity, of lysosomes (Lee J.H. et al., 2010). This effect on lysosomes is specific to the holoprotein function since γ-secretase inhibition, or loss of the essential γ-secretase complex component NICASTRIN, do not affect lysosomal acidification (Lee J.H. et al., 2010). Furthermore, EOfAD mutations have never been discovered in the genes encoding the other components of γ-secretase complexes, while mutations in three of the four genes encoding components of γ-secretase complexes (including a frame-shift mutation in *PSEN1*) cause the skin disease acne inversa and not EOfAD (Wang B. et al., 2010; Li et al., 2011; Liu et al., 2011). Also, intriguingly, another Alzheimer’s disease-related phenomenon that appears to be relatively insensitive to changes in γ-secretase activity is the increased apposition between the endoplasmic reticulum (ER) and mitochondria seen in fibroblasts from both *PSEN* EOfAD and LOsAD patients (Area-Gomez et al., 2012) [but the apposition is sensitive to β-secretase inhibition (Pera et al., 2017)]. This is important since the PSEN proteins and γ-secretase activity are highly concentrated in this apposition structure known as the mitochondria-associated membranes (MAM; Area-Gomez et al., 2009, 2012).

In a review paper published in 2016, some of us examined the evidence for the involvement of γ-secretase activity in Alzheimer’s disease (Jayne et al., 2016). We found that the genetic data from disease-causing mutations in the PSENs and other components of γ-secretase complexes supported an alternative idea. We proposed that EOfAD mutations in the PSENs promote Alzheimer’s disease through their effect on holoprotein function. Indeed, their dominant action may be due to the formation of holoprotein multimers whereby mutant holoproteins bind to, and interfere with, the action of wild type PSENs. In that paper, we conceded that this idea could not explain some reported EOfAD-related phenomena. In particular, the role of APP in this alternative view was not obvious and we had no alternative explanation for the remarkable reported correlation between the concentration ratio of Aβ_40_ relative to Aβ_42_ and the mean age of onset of EOfAD for different mutations in *PSEN1* (Duering et al., 2005).

In late 2016, Sun et al. (2017) published their comprehensive analysis of γ-secretase activity and Aβ formation for 138 different EOfAD mutations of *PSEN1*. They found that different EOfAD mutations can either increase or decrease γ-secretase activity and that the correlation of the Aβ_40_/Aβ_42_ ratio with the mean ages of onset of EOfAD mutations of *PSEN1* is illusory. Furthermore, Arimon et al. (2015) showed that changes in the ratio of Aβ_40_ relative to Aβ_42_ can occur due to oxidative stress, which is a common phenomenon in Alzheimer’s disease brains (Martins et al., 1986; Sanabria-Castro et al., 2017). Thus, in reality, there is currently little genetic data to support a role for γ-secretase (and hence Aβ) in EOfAD (other than the existence of EOfAD mutations in the γ-secretase-cleavage site of APP).

## An Alternative Link Between EOfAD-Associated Mutations in App and the PSENs

Since APP and the PSENs are linked in their common involvement in Aβ production, an alternative hypothesis for Alzheimer’s disease pathogenesis requires that a convincing alternative explanation is given for the relationship between the functions of APP and PSENs, and EOfAD pathology. While considerable effort has been devoted to understanding the relationship between EOfAD mutations and γ-secretase activity, relatively little is known about the effects of these mutations on PSEN holoprotein function or the normal functions of APP, and whether there is any commonality in function that links them. The genes *PSEN1* and *PSEN2* encode proteins with closely related structures and similar functions so it is perhaps unsurprising that EOfAD mutations should be found in both. However, APP is also part of a larger protein family. It shares structural and redundant functional activity with two other proteins, the AMYLOID BETA A4 PRECURSOR-LIKE PROTEINS 1 and 2 (APLP1, APLP2; structurally, APP is more similar to APLP1, Shariati and De Strooper, 2013). Why have EOfAD mutations never been found in the genes encoding these other proteins? What is unique about APP that is not shared with other members of its family?

Of course, of the three APP-related proteins, only APP itself can produce the Aβ peptide. Despite its close similarity to APP, the protein APLP1 apparently does not require cleavage by α- or β-secretase in order to be cleaved by γ-secretase (Schauenburg et al., 2018). However, both APLP1 (Li and Sudhof, 2004) and APLP2 (Pastorino et al., 2004) can be cleaved by β-secretase (Pastorino et al., 2004), and Aβ-equivalent peptides have been detected for these proteins (Eggert et al., 2004; Yanagida et al., 2009) although there is little to suggest that these peptides have pathological activity.

Another characteristic that APP does not share with the APLP proteins is its role in neuronal iron homeostasis. APP (and the α-secretase-cleaved secreted form of APP, sAPPα, but not APLP2) associates with FPN1 to facilitate the iron export function of FPN1 in the plasma membrane of neurons (Duce et al., 2010; Wong et al., 2014). APP’s role in iron export is so significant that it is amongst a list of other iron-related proteins (including FPN1 and others described later such as ACO2, FTH1, FTL, and SDHB (Kuhn, 2015)) whose expression is regulated post-transcriptionally by the presence of ‘iron responsive elements’ (IREs) in the untranslated regions (UTRs) of their respective mRNAs. When cellular iron levels are depleted, “IRON-RESPONSIVE ELEMENT-BINDING PROTEIN 1” (IRP1) (and related protein IRP2) are activated to bind to the stem-loop structure formed by the iron response element (IRE) motif. Whether IREs lie in the 5′ or 3′ UTR of a particular mRNA determines whether IRP binding results in translational silencing, or transcript stabilization, respectively. By regulating expression of key players in iron homeostasis, IRPs act to increase the bioavailability of iron under conditions of iron deficiency.

In the case of APP, IRP1 binds to an IRE motif in the 5′ UTR of *APP* mRNA to inhibit translation (Cho et al., 2010) and thus inhibit iron export (Rogers et al., 2002) in iron deficiency. This motif is not present in the 5′ UTR of *APLP1* (Rogers et al., 2002) or *APLP2* (Maloney et al., 2004).

In iron replete conditions, IRP1 incorporates an iron–sulfur cluster (ISC) to become enzymatically active as cytosolic protein ACONITASE1 (ACO1; related to the mitochondrial ACONITASE2 protein required by the tricarboxylic acid cycle, TCA, for energy production) (Hentze and Argos, 1991; Rouault et al., 1991). In this form, it is unable to bind IRE sequences. Notably, ACO1 may possibly also be converted to IRP1 under conditions of oxidative stress (Gehring et al., 1999), e.g., due to either hypoxia or hyperoxia (Terraneo and Samaja, 2017). Analysis of the subcellular localization of IRP1 shows it to be concentrated in the ER (Patton et al., 2005), which is consistent with the importance in Alzheimer’s disease of the MAM (see later).

The importance of APP in iron homeostasis is demonstrated by the structural similarity of its 5′ UTR sequence to those of FERRITIN LIGHT CHAIN (FTL) and FERRITIN HEAVY CHAIN 1 (FTH1) (Rogers et al., 1999), two proteins that together form the FERRITIN molecular cages in which iron is stored as Fe^3+^. Like APP, translation of the ferritin subunits is inhibited by IRP-IRE binding (to decrease iron storage when iron levels are depleted). Note that, unlike the 5′ UTR of *FTH1* mRNA, *APP* mRNA’s 5′ UTR may not bind IRP2 (Cho et al., 2010). IRP2 is structurally similar to IRP1 (Rouault et al., 1990) but lacks aconitase activity (Guo et al., 1994; Samaniego et al., 1994) and becomes unstable when cellular iron levels are high (Iwai et al., 1995) (rather than becoming, like IRP1, unable to bind IREs due to possession of an iron–sulfur cluster).

Translation of *APP, FTL*, and *FTH1* mRNAs are also all apparently stimulated by the binding of the pro-inflammatory cytokines INTERLEUKIN 1 ALPHA (IL1A) or INTERLEUKIN 1 BETA (IL1B) to INTERLEUKIN translation enhancer elements in their 5′ UTRs. In fact, in the case of FTH1, iron and IL1B act synergistically to increase translation of *FTH1* mRNA (Thomson et al., 2005). Thus, APP, FTL, and FTH1 proteins all participate in the acute phase inflammatory response. FTL and FTH1 upregulation acts to sequester iron in FERRITIN making it unavailable to invading bacteria (Wessling-Resnick, 2010; Soares and Weiss, 2015). The upregulation of APP by inflammatory cytokines suggests that, in inflammation, there may be a need to export iron from the cytosol of cells that express APP, which in the brain is specifically neurons (and not glia or astrocytes) (Guo et al., 2012). APP’s role in the acute phase inflammatory response is discussed later. The regulation of APP mRNA translation has been reviewed by Ruberti et al. (2010) and some aspects of this are illustrated in **Figure 1**. The translation of APP mRNA is also regulated by FRAGILE X MENTAL RETARDATION PROTEIN (FMRP, encoded by gene *FMR1*) through its binding to a G-quartet-like RNA motif within the APP coding sequence (Westmark and Malter, 2007; Lee E.K. et al., 2010). Binding of other proteins, particularly in the 3′ UTR, regulates APP mRNA stability (e.g., Zaidi et al., 1994; Zaidi and Malter, 1995 and see reviews Ruberti et al., 2010; Westmark and Malter, 2012).

Interestingly, the trafficking of APP to the plasma membrane where it can interact with FPN1 is dependent on the activity of MAPT (Lei et al., 2012, 2017). Consequently, mice lacking MAPT expression show accumulation of iron in the brain (Lei et al., 2012). Apparently, iron can both induce MAPT hyperphosphorylation (Xie et al., 2012; Guo et al., 2013) and encourage aggregation of hyperphosphorylated MAPT (Yamamoto et al., 2002). Since MAPT becomes hyperphosphorylated and aggregates in the neurons of Alzheimer’s disease brains, we would expect this to reduce the available APP on the neuronal plasma membrane and so reduce iron export in aged neurons showing Alzheimer’s disease pathology. (Note the potential here for a positive feedback loop driving iron accumulation.)

Gene knockout studies in mice also point to the impact of age on APP’s effect on iron homeostasis. While mice lacking APP apparently show reduced body weight when young (Zheng et al., 1995), they do not show excess brain iron accumulation (compared to wild type) by 3 months of age. However, brain iron accumulation is significantly increased by 12 months of age (Needham et al., 2014). In contrast, mice lacking APLP2 show no difference in brain iron accumulation compared to wild type mice at any age (Needham et al., 2014). The age-dependence of iron accumulation due to APP dysfunction in mice is consistent with both familial and sporadic Alzheimer’s disease as adult onset diseases in humans. Apparently, neurons (and any other cells in which APP may function in iron export) are not critically dependent on this function during early development. In fact, iron accumulation is important for early brain development and insufficient early brain iron accumulation may determine an aberrant iron “set point” that is refractory to alteration by later iron supplementation (Hare et al., 2013).

Like APP (and as described later in this paper), the PSENs can be expected to play a role in iron homeostasis in neurons (and other cells) due to their role in acidification of the endo-lysosomal pathway (Lee J.H. et al., 2010; Lee et al., 2015). We propose that a shared function of EOfAD-associated proteins may be normal roles in regulation of neuronal iron homeostasis, particularly as animals age.

## Cellular Iron Homeostasis and the EOfAD Mutations of *App*

Do the characteristics of *APP*’s EOfAD mutations support that APP’s role in iron homeostasis is critical for Alzheimer’s disease pathogenesis? There are two broad categories of EOfAD mutations affecting *APP*: (1) whole gene duplications and, (2) missense mutations (see the review by Tcw and Goate, 2017). Both categories of *APP* EOfAD mutations preserve the ability to produce a full-length APP protein (similar to EOfAD mutations in the *PSEN* genes). Individuals with trisomy of chromosome 21 possess three copies of the *APP* gene and show early onset Alzheimer’s disease (Prasher et al., 1998). A number of the missense mutations in APP might also be expected to increase the stability (and effective dosage) of APP expression. For example, mutations that inhibit cleavage of APP by α-secretase are suggested to be pathogenic by their enhancement of β-secretase cleavage to form Aβ rather than the shorter, non-pathogenic p3 peptide formed by α-secretase cleavage (Haass et al., 1994; Kaden et al., 2012). However, these mutations should also increase the stability of full-length APP, particularly when one remembers that cleavage of APP by β-secretase occurs mainly at the β′ site (**Figure 1**) rather than the β-site cleavage that produces Aβ during cellular stress (Deng et al., 2013). Similarly, even though γ-secretase cleavage of APP is thought to occur subsequent to α-secretase or β-secretase cleavage, there is evidence that these latter two enzymes act in complexes together with γ-secretase (Teng et al., 2010; Chen et al., 2015; Cui et al., 2015) suggesting that inhibition of γ-secretase might also lead to inhibition of initial cleavage by α- or β-secretases. Xu et al. (2016) recently showed that most EOfAD mutations in APP decrease the efficiency of its cleavage by γ-secretase. An effective over-expression of full-length APP would be expected to result in excessive Fe^2+^ export from neurons leading to a deficiency of cytosolic Fe^2+^. On the other hand, mutations in APP that increase β-secretase cleavage (such as the “Swedish” mutation, KM670/671NL, Citron et al., 1992) could lead to deficient Fe^2+^ export and neuronal iron accumulation.

**FIGURE 2:**
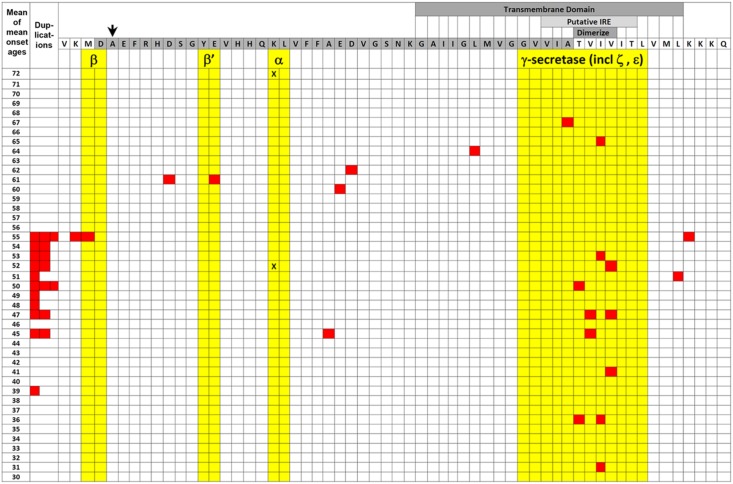
EOfAD mutations of APP. The transmembrane domain (labeled darker gray box) and Aβ region (darker gray-shaded amino acid residues) of APP protein are illustrated relative to the mean of mean onset ages (MMOA) of mutations (red squares) from the *Alzheimer Disease & Frontotemporal Dementia Mutation Database* (Cruts et al., 2012). MMOAs for duplications of the *APP* gene are shown on the left hand side. There is no MMOA for the K687N mutation in the α-secretase cleavage site but the two onset ages published (Kaden et al., 2012) are indicated by black “X”s. An arrow indicates the position of the protective A673T mutation that reduces Aβ production (Jonsson et al., 2012). Cleavages by various secretases occur between amino acid residues colored yellow. Positions of the possible downstream IRE (see also **Figure 3**) and the amino acid residues required for dimerization are also shown by labeled lighter and darker gray boxes, respectively. A two-tailed *t*-test assuming unequal variances for differences between the means of all the MMOAs of whole gene duplications versus non-γ-secretase-affecting missense mutations gives *p* = 0.05 suggesting that the mechanisms by which these mutation classes generate pathology may differ. We note that this data is also consistent with the suggestion by Area-Gomez et al. (2018) that increased formation of the β-secretase-cleaved transmembrane fragment of APP (the C99 fragment) is the critical effect of EOfAD mutations in APP. C99 accumulates in the MAM and appears to mediate the effects of PSEN activity on MAM formation with consequences for energy metabolism (Pera et al., 2017). EOfAD mutations of APP also alter the tendency of Aβ to aggregate (Hatami et al., 2017) but we know of no correlation between Aβ aggregation dynamics and MMOA.

The α-secretase-cleaved secreted form of APP, sAPPα, can facilitate FPN1 activity in a manner similar to full-length APP (Wong et al., 2014). This raises the important question of the activity of the β-secretase-cleaved secreted form, sAPPβ. An inability of sAPPβ to facilitate FPN1 activity would support the idea that iron homeostasis is the critical function affected by EOfAD mutations in APP. sAPPα and sAPPβ differ by only 16 amino acid residues in length and have largely similar functions although distinct differences in activity have also been observed (reviewed by Ludewig and Korte, 2016; Mockett et al., 2017). For example, expression of sAPPα, but not sAPPβ, can rescue the viability of mice that simultaneously lack both APP and APLP2 (Ring et al., 2007; Li et al., 2010; Weyer et al., 2011). [This shows that much of the activity of full-length APP can be attributed to sAPPα (Ring et al., 2007; Weyer et al., 2011)]. sAPPα can also facilitate long term potentiation in the hippocampus (Taylor et al., 2008; Hick et al., 2015) in contrast to sAPPβ (Hick et al., 2015). Chen et al. (2017) and Peters-Libeu et al. (2015) showed that sAPPα can inhibit the activity of β-secretase suggesting the existence of a “molecular switch” where one secretase pathway inhibits the other. Importantly, the latter study used small angle X-ray scattering to model the tertiary structures of sAPPα and sAPPβ and showed them to be very different. This was supported by fluorescence spectroscopy data (Peters-Libeu et al., 2015). However, the relative abilities of sAPPα and sAPPβ to stabilize FPN1 are yet to be tested. Further complicating this issue is the fact that, in the absence of cellular stress, most β-secretase cleavage of APP occurs at the β′ site 10 amino acid residues downstream of the β site (Deng et al., 2013) (**Figure 2**). Nevertheless, the activity of sAPPβ′ has been largely overlooked by researchers.

An important possible implication of the ability of sAPPα to facilitate FPN1 activity is that increased gene dosage of APP (e.g., due to duplication mutations or in Down syndrome) could potentially cause excess sAPPα secretion from neurons that might act to stabilize FPN1 on the plasma membranes of proximal cells. Indeed, an elegant gene-trap experiment described by Liao et al. (2012) showed that only neurons in the developing brains of zebrafish embryos express APP but that APP’s extracellular domain accumulates in brain vasculature. Therefore, the potential may exist for non-autonomous cellular dyshomeostasis of iron due to changes in the expression and cleavage of APP in neurons. Interestingly, a common pathology of *APP* duplications (Cabrejo et al., 2006) and Down syndrome (Donahue et al., 1998; Buss et al., 2016) is intracerebral hemorrhage suggesting that increased APP expression disrupts the integrity of brain vasculature. Accumulation of Aβ in brain vasculature is also very common in Alzheimer’s disease [i.e., cerebral amyloid angiopathy (CAA; Vinters and Gilbert, 1983; Attems, 2005)] and probably contributes to this disruption (Samuraki et al., 2015) with the consequent release of heme contributing to higher iron levels in Alzheimer’s disease brains.

A recent paper by Lopez Sanchez et al. (2017) illustrated how different EOfAD mutations of APP might have differing, but still detrimental, effects on iron homeostasis. Forced over-expression of full-length wild type APP in human SH-SY5Y neuroblastoma cells led to decreased mitochondrial respiration and increased glycolysis (lactate production). This would be expected since the predicted cytosolic Fe^2+^ deficiency caused by APP overexpression would deprive the mitochondrial TCA and electron transport chain of iron–sulfur clusters while stabilizing the HIF1 transcription factor that regulates the hypoxia response (see later). In contrast, forced over-expression of the EOfAD “Swedish mutation” form of APP that shows enhanced β-secretase cleavage did not decrease mitochondrial respiration (despite increased production of Aβ_42_) unless β-secretase was inhibited, in which case it had the same effect as wild type APP. This supports that different EOfAD mutations of APP have different effects on energy metabolism, possibly as a result of differential effects on cytosolic Fe^2+^ availability. [The situation is further complicated by the observation of Chen et al. (2017) that iron can inhibit β-secretase activity while Bodovitz et al. (1995) noted that high iron levels can increase α-secretase activity.] In fact, examining the “mean of mean onset ages” (MMOA) of different EOfAD mutations in APP from the *Alzheimer Disease & Frontotemporal Dementia Mutation Database* (Cruts et al., 2012) shows that missense mutations (excluding those thought to affect γ-secretase cleavage) such as the Swedish mutation tend to cause later onset ages than duplications of *APP* (i.e., they are less pathogenic, **Figure 2**).

And how should we interpret the “Alzheimer’s disease-protective” mutation of APP, A673T? Icelandic carriers of this mutation show decreased risk of Alzheimer’s disease and increased cognitive performance when elderly (Jonsson et al., 2012). The allele is rare outside of Iceland (Kero et al., 2013; Ting et al., 2013; Bamne et al., 2014; Mengel-From et al., 2015; Wang L.S. et al., 2015). A673T is claimed to reduce cleavage at the β-site to reduce Aβ synthesis (Jonsson et al., 2012). Since cleavage at the β-site of APP is a stress response (particularly to infection – see later) and suppression of inflammatory stress is protective of aging vasculature (Ganjehei and Becker, 2015; Wang J. et al., 2015), it may be that A673T acts to reduce chronic inflammatory stress levels and preserve vascular health. Intriguingly, the Icelandic population (particularly the rural population living under conditions more similar to those prevailing for most of Iceland’s millennium of settlement), shows unusually high levels of serum FERRITIN (Jonsson et al., 1991). It is possible that an increased rate of iron export from neural cells is protective (i.e., advantageous in natural selection) under such conditions.

**FIGURE 3:**
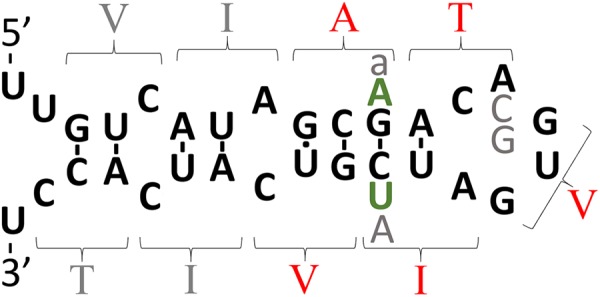
The intriguing story of APP’s possible downstream IRE. The theoretical secondary structure of the possible downstream IRE originally published by Tanzi and Hyman (1991) shortly before the Alzheimer’s disease field became focused on the idea that production of Aβ_42_ is critical to Alzheimer’s disease pathogenesis. The amino acid residues specified by codons are indicated by parentheses. Those amino acid residues shown in red are mutated in EOfAD. In 1991, it was suggested that transmembrane domain EOfAD mutations in APP might exert their pathological effect by disrupting regulation of APP by iron (and this was before APP’s role in iron homeostasis was known). However, the discovery in the following year of an apparently non-pathological (but theoretically IRE-disruptive) polymorphism in this region (Zubenko et al., 1992) (gray “A”) collapsed support for this suggestion. Most IREs are found either in the 5′- or 3′-UTRs of mRNAs where binding of IRPs inhibits or enhances, respectively, mRNA translation (Casey et al., 1988; Anderson et al., 2012). The position of APP’s putative, second IRE within transmembrane domain-coding sequences is unexpected and there is no experimental data supporting its functional reality. Rather, binding studies suggest that, unlike the IRE in APP’s 5′ UTR, this second, IRE-like structure does not bind IRP1 (Cho et al., 2010). However, it is intriguing that in the most diverged (from humans) animal known to possess an Aβ sequence, the Coelacanth, and in most placental mammals, there are two nucleotide differences in the area of the putative IRE and these are conservative since they would preserve base pairing in a double helical region of this structure (green “A” and “U,” found by inspection of genome sequences available at ensembl.org). Other variations found are: gray “a,” an alternative nucleotide in olive baboon and macaque; gray “C” and “G,” alternative nucleotides observed in mouse and kangaroo rat, respectively. If real, one possibility might be that this IRE-like structure only binds an IRP in co-operation with other protein(s) and that it controls mRNA stability. Alternatively, the two IRE’s of APP may interact via co-operative binding of IRPs, an idea alluded to by E. C. Theil (Silver and Walden, 1998) and consistent with formation of an IRP1 complex that can bind two IREs (Hu and Connor, 1996) but never tested for APP’s mRNA.

An additional complicating factor in considering how EOfAD mutations might affect iron homeostasis is our current limited knowledge regarding how FPN1 and APP interact. For example, APP can dimerize and transmembrane domain amino acid residues critical for this (and affecting γ-secretase cleavage) are mutated in EOfAD (Yan et al., 2017) (**Figure 2**). The dimerization of APP affects its interaction with the protein SORL1 and this influences the trafficking of APP from the ER to the plasma membrane (Eggert et al., 2017) where FPN1 acts to export Fe^2+^. The most severe EOfAD mutations in APP (those with the earliest Alzheimer’s disease onset ages) affect the transmembrane domain amino acid residues critical for dimerization (**Figure 2**). Intriguingly, the region of *APP* RNA that codes for these transmembrane amino acid residues is also predicted to fold into a possible second, downstream IRE (**Figure 3**).

## Cellular Iron Homoeostasis and the PSEN Proteins

**FIGURE 4:**
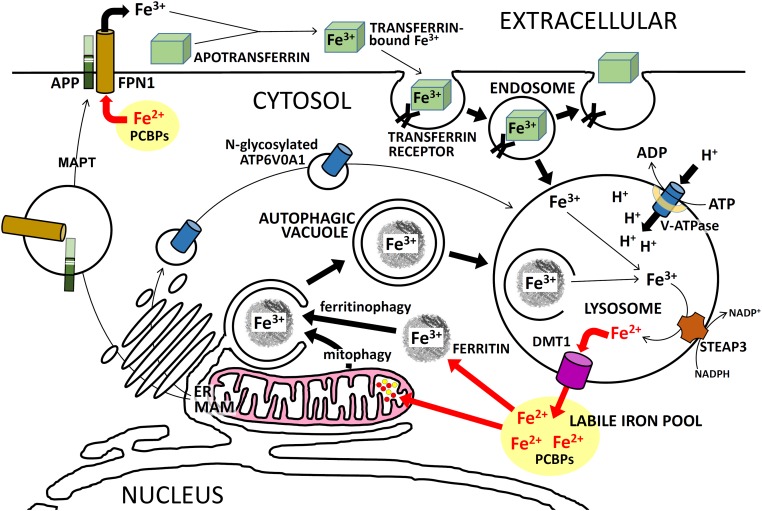
Intracellular iron trafficking in neurons. A simplified description of the trafficking of iron ions within neurons, showing the importance of APP and PSEN for these functions. PSENs (in the MAM of the ER) are required for normal *N*-glycosylation of ATP6V0A1 which is an essential component of the vacuolar ATPase (v-ATPase). Failure to sufficiently acidify endosomes/lysosomes retards Fe^3+^ release from TRANSFERRIN/TFRC complexes and should retard recycling of Fe^3+^ to Fe^2+^ via autophagy. This can cause iron to accumulate in cells as Fe^3+^. Excessive full-length APP may cause excessive FPN1 activity which would lower Fe^2+^ levels in the cytosol. Activity of MAPT (tau) is required for transport of APP to the plasma membrane. See the text for further explanation.

The probable dysregulation of cytosolic Fe^2+^ homeostasis due to EOfAD mutations in APP is significant because EOfAD mutations in the PSENs are predicted to produce similar effects. The major route of iron entry into cells is via endosomes/lysosomes. TRANSFERRIN-Fe^3+^ complexes bind to TRANSFERRIN RECEPTOR on the surface of cells for endocytosis into endosomes (reviewed in Mayle et al., 2012). In the endosomal-lysosomal system, acidification by v-ATPase causes release of Fe^3+^ from TRANSFERRIN (Pantopoulos, 2004) and then the Fe^3+^ is reduced to the biologically active form, Fe^2+^, by SIX-TRANSMEMBRANE EPITHELIAL ANTIGEN OF PROSTATE 3 (STEAP3) (Passer et al., 2003; Grunewald et al., 2012). As stated earlier, PSEN1 is required for acidification of lysosomes (and, presumably, endosomes) by facilitating *N*-glycosylation of ATP6V0A1 (**Figure 4**).

After formation, Fe^2+^ is transported out of endocytic vacuoles and into the cytosol by the protein SOLUTE CARRIER FAMILY 11 [PROTON-COUPLED DIVALENT METAL ION TRANSPORTER], MEMBER 2 (SLC11A2, more commonly known as DIVALENT METAL TRANSPORTER 1, DMT1) (Moos and Morgan, 2004). Other proteins that can transport Fe^2+^ out of lysosomes are MUCOLIPIN 1 (MCOLN1; Dong et al., 2008, previously known as TRPML1) and SLC11A1 (Evans et al., 2001) (previously named NRAMP1).

## The Cytosolic Labile Iron Pool, Ferritin, and Autophagy

The nature of the cytosolic “labile iron pool” (simple iron ions and iron–sulfur clusters) has not been well characterized. Due to its highly reactive nature, free Fe^2+^ most likely does not occur in cells under normal conditions. Instead, this ion may always be chaperoned as it is distributed to the many proteins that require it for activity. Cytosolic Fe^2+^ can be chaperoned by POLY(rC)-BINDING PROTEINs (PCBPs) to, either, FERRITIN cages (Shi et al., 2008) (where it is re-oxidized to Fe^3+^ for storage), or to mitochondria [where it is imported via MITOFERRIN and other proteins (Richardson et al., 2010)], or to cytosolic target proteins (see recent reviews by Lane et al., 2015 and Philpott et al., 2017 and see **Figure 4** and below). [2Fe-2S] iron–sulfur clusters appear to be chaperoned in the cytosol by complexes of the proteins GLUTAREDOXIN 3 (GLRX3) and BolA, E. COLI, HOMOLOG OF, 2 (BOLA2, reviewed by Philpott et al., 2017).

POLY(rC)-BINDING PROTEINs not only chaperone cytosolic Fe^2+^ to FERRITIN but also regulate FERRITIN synthesis through control of *FTH1* mRNA translation. PCBP1 and PCBP2 have been observed to bind to the same stem-loop structure in the 5′ UTR of *FTH1* mRNA as does IL1 (Thomson et al., 2005). The similarities between the 5′ UTRs of FERRITIN and APP mRNAs lead us to speculate that PCBPs may also regulate translation of APP although this is yet to be demonstrated experimentally.

In order to utilize the cytosolic Fe^3+^ stored in FERRITIN cages it must be reduced once again to Fe^2+^ within lysosomes after delivery by autophagy (Mancias et al., 2014). Autophagy of FERRITIN, or “ferritinophagy,” requires the specific cargo receptor protein NUCLEAR RECEPTOR COACTIVATOR 4 (NCOA4) (Dowdle et al., 2014; Mancias et al., 2014), expression of which is regulated by iron. High iron levels destabilize NCOA4 to slow the recycling of Fe^3+^ to Fe^2+^ via ferritinophagy (Mancias et al., 2015). Notably, autophagy was recently shown to initiate at the MAM (Hamasaki et al., 2013), a structure that also appears to regulate mitochondrial activity [by Ca^2+^ release (Carreras-Sureda et al., 2017)]. The MAM also coordinates EOfAD protein function (Area-Gomez et al., 2009), and is involved in Aβ production (discussed in Jayne et al., 2016), and in the initiation of inflammation (Misawa et al., 2013). The MAM of yeast was recently shown to be necessary for iron homeostasis (Xue et al., 2017) although whether this is conserved in humans is unknown. The autophagy of mitochondria, or “mitophagy,” is also an important part of cellular iron homeostasis since these organelles contain so much of the element. The necessity of autophagy for recycling of inactive, stored Fe^3+^ into active Fe^2+^ means that a cell with defective autophagy or deficient lysosomal acidification might accumulate iron to high levels while suffering an effective deficiency of cytosolic Fe^2+^. A similar situation occurs in the recessively inherited disorder mucolipidosis IV (ML4) where loss of *MCOLN1* activity results in accumulation of Fe^2+^ in lysosomes with simultaneous cytosolic Fe^2+^ deficiency (Dong et al., 2008). This results in degeneration of retinas, motor impairment and intellectual disability (Dong et al., 2008).

The *APOE* gene that is the major locus for variation affecting risk of LOsAD (Tanzi, 2012) was recently shown to play a role in the transcriptional regulation of autophagy (Parcon et al., 2017; Theendakara et al., 2017). The Alzheimer’s disease-pathogenic 𝜀4 allele of *APOE* (*APOE4*) caused significantly lower levels of particular autophagy-critical gene transcripts compared to the non-pathogenic 𝜀3 allele (*APOE3*) both in Alzheimer’s disease brains and in a transfected astroglioma cell line (Parcon et al., 2017).

Finally, Aβ has been shown to bind Fe^2+^ (Bousejra-ElGarah et al., 2011; Boopathi and Kolandaivel, 2016). This means that accumulation of Aβ might sequester Fe^2+^ and contribute to a cytosolic deficiency, and is consistent with Aβ’s speculated antibacterial role (see later). Indeed, this might explain why one candidate anti-Aβ therapy has been seen to have ameliorative effects (even if somewhat minor) on Alzheimer’s disease progression in a phase 1b clinical trial (Sevigny et al., 2016, 2017).

## The Intimate Relationship Between Iron Homeostasis, Energy Metabolism, and Responses to Hypoxia

Many proteins require Fe^2+^ as a cofactor, especially mitochondrial proteins involved in the TCA (Mailloux et al., 2007) (mitochondrial ACONITASE [ACO2], and SUCCINATE DEHYDROGENASE COMPLEX, SUBUNIT B, IRON SULFUR PROTEIN [SDHB]) and in the protein complexes of the electron transport chain (reviewed by Stiban et al., 2016). Therefore, a deficiency of cytosolic Fe^2+^ is expected to interfere with normal mitochondrial function and increase oxidative stress (Walter et al., 2002; Xu et al., 2013) while excessive cytosolic iron accumulation is thought to cause oxidative stress via Fenton chemistry (Winterbourn, 1995) (below). This is consistent with the fact that aberrant mitochondrial activity is a common phenomenon observed in Alzheimer’s disease studies (reviewed in Silva et al., 2012).

Electron transport chains causing reduction of oxygen (O_2_) exist both in mitochondria (as part of oxidative phosphorylation) and in the MAM of the ER [involved in the oxidative protein folding that forms disulfide bonds in proteins (Riemer et al., 2009)]. Both processes produce considerable quantities of H_2_O_2_ that can interact, in Fenton chemistry, with either Fe^2+^ or Fe^3+^ to produce highly reactive hydroxyl (HO^•^) or hydroxyperoxyl (HOO^•^) radicals, respectively (Wardman and Candeias, 1996). Oxidative stress that exceeds mitochondria’s antioxidant capacity will damage iron-containing proteins leading to additional iron release in a potential positive feedback loop. This further decreases mitochondrial function and cells’ capacity for production of energy by oxidative phosphorylation (Mandelker, 2008).

The critical dependence of mitochondria on both iron and oxygen for energy production is demonstrated by the intimate coupling of cellular responses to iron or oxygen deficiency. The concentration of Fe^2+^ is central to controlling the stability of the protein HYPOXIA-INDUCIBLE FACTOR 1, ALPHA SUBUNIT (HIF1α) that, together with its partner ARYL HYDROCARBON RECEPTOR NUCLEAR TRANSLOCATOR (ARNT, also known as HIF1β) forms the transcription factor HIF1, a master regulator of cellular responses to hypoxia. Low cytosolic Fe^2+^ concentrations lead to stabilization of HIF1α by inhibiting the activity of EGLN proteins (“EGL9, C. ELEGANS, HOMOLOG OF,” previously designated as the PHD proteins). EGLNs are Fe^2+^-dependent prolyl hydroxylases that act on HIF1α to stimulate its degradation (Bishop and Ratcliffe, 2015). Significantly, because HIF1 activity is sensitive to changes in cytosolic Fe^2+^ availability, this transcription factor also plays a role in cellular iron homeostasis by regulating the expression of numerous genes/proteins involved in iron trafficking including TRANSFERRIN, TRANSFERRIN RECEPTOR, SLC11A2, and FPN1 (see review by Lane et al., 2015).

The involvement of HIF1 in Fe^2+^ homeostasis is additionally significant since the EOfAD genes all show strong upregulation by hypoxia (Moussavi Nik et al., 2012 and references therein) and there is a great deal of circumstantial evidence implicating hypoxia as an important component in LOsAD (Raz et al., 2016). However, the PSENs are not simply targets of HIF1 regulation. Instead, they seem to be central to the activity of HIF1. Using immortalized mouse fibroblasts, De Gasperi et al. (2010) showed that Psen1 binds Hif1α and is required for normal Hif1α stabilization. Intriguingly, Hif1α stability can also be regulated by insulin (De Gasperi et al., 2010) providing a possible link between cytosolic Fe^2+^ and type II diabetes, a recognized risk factor for Alzheimer’s disease (Jayaraman and Pike, 2014). Dramatic, age-dependent changes in the ability to stabilize Hif1α under hypoxia have been seen in rat brains (Ndubuizu et al., 2009) while Liu et al. (2008) observed low levels of HIF1α in LOsAD brains compared to aged controls.

HIF1 plays a central role in regulation of energy metabolism since this transcription factor directly induces transcription of *PYRUVATE DEHYDROGENASE KINASE, ISOENZYME 1* (*PDK1*) (Kim et al., 2006). The PDK1 protein phosphorylates the PYRUVATE DEHYDROGENASE COMPLEX, ALPHA-1 subunit (PDHA1) of the PYRUVATE DEHYDROGENASE COMPLEX thereby inhibiting the conversion of pyruvate to acetyl-CoA. This means that, under hypoxia, more of a cell’s glucose budget is directed away from the TCA and oxidative phosphorylation and into ATP production by the less efficient (but more rapid) process of anaerobic glycolysis. This maintains cellular ATP levels while protecting cells from the high levels of reactive oxygen species (ROS) that are generated by oxidative phosphorylation performed under low oxygen (Zhang et al., 2008). Deficient cytosolic Fe^2+^ levels would also be expected to stabilize HIF1α and skew cellular energy metabolism toward glycolysis, in essence imitating hypoxic stress. Here, we see a possible overlap with LOsAD since changes in vasculature are observed earlier than Aβ accumulation in development of LOsAD (Iturria-Medina et al., 2016) and it is well established that blood flow in Alzheimer’s disease brains is decreased (Farkas and Luiten, 2001; Marlatt et al., 2008; Nicolakakis and Hamel, 2011; Daulatzai, 2017).

## An Overload of Cellular Iron Can Disguise a Cytosolic Fe^2+^ Deficiency

Because of iron’s central cellular role in energy metabolism, it is unsurprising that many neurological and neurodegenerative conditions show iron dysregulation (see recent excellent reviews, Mills et al., 2010; Lane et al., 2015; Meyer et al., 2015; Gozzelino and Arosio, 2016). The accumulation of iron with age appears to be a universal phenomenon in animals (Massie et al., 1985) and most neurodegenerative disorders apparently involve excess iron in the brain. Excess iron can promote Fenton chemistry that causes oxidative stress by production of hydroxyl radicals (Greenough et al., 2013). Significantly, oxidative stress is thought to stabilize HIF1α by inhibiting EGLN activity (Page et al., 2008). Therefore, iron accumulation should disturb the balance between glycolysis and oxidative phosphorylation, especially if age/pre-existing metabolic stress inhibits the production of antioxidants (Zhu et al., 2006).

Because the subcellular location and form of iron is critical to its function, it is possible to have a cytosolic deficiency of Fe^2+^ while accumulating abnormally high levels of iron in the endolysosomal compartment. As mentioned previously, this occurs in the neurodegenerative condition Mucolipidosis IV when loss of function of MCOLN1 inhibits transport of Fe^2+^ into the cytosol from endosomes and lysosomes (Dong et al., 2008). Similarly, we can predict that a failure to sufficiently acidify endolysosomes and lysosomes (such as due to EOfAD mutations in the *PSEN* genes) should inhibit importation of Fe^2+^ into the cytosol as well as the recycling of cytosolic Fe^3+^ stored within FERRITIN cages to Fe^2+^ via autophagy (and the recycling of iron in mitochondria via mitophagy). Indeed, this may occur in a juvenile onset form of autosomal recessive hereditary parkinsonism, Kufor-Rakeb syndrome, where loss of function of *ATPase, TYPE 13A2* (*ATP13A2*) results in iron accumulation and neurodegeneration (Dehay et al., 2012; Usenovic et al., 2012; Meyer et al., 2015). There are many parallels between the pathologies of Alzheimer’s disease and Parkinson disease (PD) including effects on mitochondrial function and energy metabolism (Calderone et al., 2016). Indeed, there are striking parallels between *APP* and the major locus for familial PD, *SYNUCLEIN, ALPHA* (*SNCA*). Both genes encode proteins that contribute to protein inclusions in neurodegenerative disease, both show localization in the MAM (Area-Gomez et al., 2009; Guardia-Laguarta et al., 2014), and both have transcripts with 5′ UTR IREs that inhibit translation when iron levels are low (Febbraro et al., 2012). In fact, the SNCA protein appears to act as a ferrireductase that can convert Fe^3+^ to Fe^2+^ in the presence of Cu^2+^ and NADH (Davies et al., 2011) and it may also be involved in the uptake of TRANSFERRIN-bound Fe^3+^ (Baksi et al., 2016).

Iron accumulates in the brain with age (Pirpamer et al., 2016) and this accumulation may be enhanced in Alzheimer’s disease brains (Schrag et al., 2011). However, this may disguise an effective Fe^2+^ deficiency in the cytosol and mitochondria. The failure of autophagic flux observed in both LOsAD brains (Nixon et al., 2005; Nixon, 2007) and in fibroblasts from individuals with EOfAD due to *PSEN1* mutations (Lee J.H. et al., 2010) implies an inability to recycle iron held within defective mitochondria and/or stored in FERRITIN. Likewise, mutations causing overexpression of full-length APP or sAPPα could cause excessive Fe^2+^ export even as Fe^3+^ accumulates due to ineffective autophagy and this will degrade mitochondrial performance leading to oxidative stress and decreased energy production.

Energy production is the central and most important cellular activity that makes possible all other activities. Falling energy production probably explains the increased tendency for proteins to aggregate with age (Cohen et al., 2006) [since cellular ATP levels decrease with age and ATP was recently shown to act as a hydrotrope that increases the general solubility of proteins (Patel et al., 2017)]. Failing cellular energy production will also affect the production of the reducing agent NADPH required for the conversion of Fe^3+^ to Fe^2+^ in lysosomes. Failing cellular energy production also means decreased production of the ATP required for synthesis of glutathione and other antioxidants. The accumulation of iron in an environment subject to oxidative stress (such as the aging brain) is potentially hazardous since ROS may disrupt iron-bearing proteins to release more iron in a positive feedback loop (Mills et al., 2010).

## Non-Neuronal Cells Play Important Roles in Brain Iron Homeostasis

Another important factor to consider in understanding the role of iron homeostasis in Alzheimer’s disease is the roles that non-neuronal cell types play in iron trafficking and storage (reviewed in Belaidi and Bush, 2016). The arguments presented in this paper are centered on iron homeostasis in neurons. However, the highest concentrations of iron in the adult brain appear to be in oligodendrocytes (Connor et al., 1990) and it is known that iron deficiency has marked effects on myelin formation (Beard et al., 2003). Also, microglia [that are markedly dystrophic in Alzheimer’s disease (Streit et al., 2009) and are regarded as macrophages of the brain] show many-fold higher iron content than neurons or astrocytes (Urrutia et al., 2013). Interestingly astrocytes have emerged as a source of HAMP, when stimulated by inflammatory mediators secreted from activated glial cells (You et al., 2017). Furthermore, inflammatory triggers (lipopolysaccharide, cytokines TNFα and IL-6), have differential effects on iron sequestration, and on the expression of genes encoding FPN1 and HAMP, in neurons, astrocytes, and microglia (Urrutia et al., 2013). Understanding how EOfAD mutations affect iron homeostasis in, and between, different neural cell types will be critical to understanding the role of iron homeostasis in Alzheimer’s disease and requires much additional investigation.

## A “Stress Threshold Change of State” Into Alzheimer’s Disease?

**FIGURE 5:**
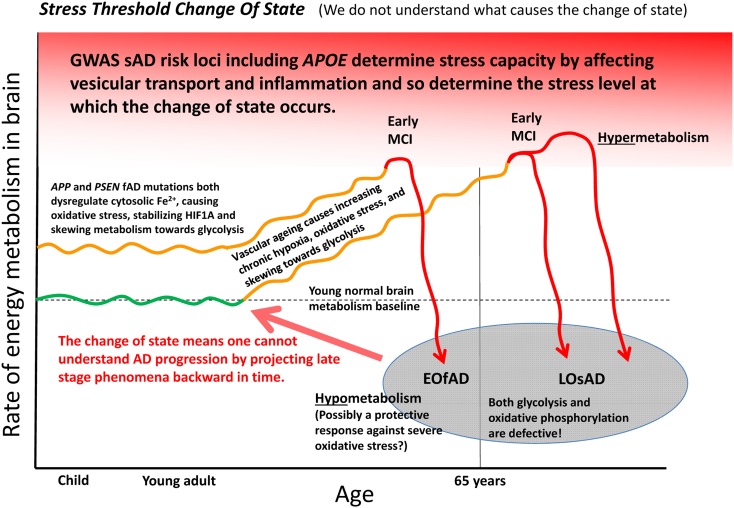
A model of Alzheimer’s disease development encompassing both EOfAD and LOsAD genetics. An early onset age of Alzheimer’s disease may occur due to metabolic stress on the brain, possibly due to iron dysregulation. This, in its effects, resembles hypoxic stress and supplements the gradually rising hypoxic stress normally driven by vascular aging so that a stress tolerance (homeostasis capacity) threshold is exceeded sooner, after which the brain shifts into a hypometabolic state, possibly in order to delay further damage due to rising oxidative stress. The timing and extent of this process will vary across different brain regions depending on basal metabolic load, glycolytic capacity, oxygen supply, etc.

Can the many functions and interactions of the EOfAD genes and iron be crystalized into an explanation of the development of Alzheimer’s disease? We suggest that one unifying mechanistic scheme may involve an interplay between aging vasculature, hypoxia, iron, and energy production. Increasing hypoxia is a characteristic of normal aging in brains as indicated by rising levels of transcription of *HIF1α* and HIF1-responsive genes with age (Lu et al., 2004). This increasing hypoxia should increasingly skew metabolism toward glycolysis. However, at some point the aging brain can undergo a shift into a hypometabolic, dysfunctional state as seen in functional MRI analyses (Ashraf et al., 2015) and as evidenced by transcriptome-level comparisons of tissue from normal aged brains, MCI brains and brains from individuals with Alzheimer’s disease (Berchtold et al., 2014). We suggest the possibility that EOfAD mutation-driven cytosolic Fe^2+^ dysregulation may cause premature utilization of a proportion of the brain’s glycolytic capacity that would otherwise serve to cope with the rising hypoxia caused by the natural decline in function of cerebral vasculature with age. Eventually, a trigger such as an infection (Mawanda and Wallace, 2013), heart failure (Cermakova et al., 2015) or acute hypoxia event (Chen et al., 2014) may force the brain over a stress threshold causing a change of state into the levels of inflammation and hypometabolism that characterize Alzheimer’s disease. This stress threshold is exceeded sooner in the pre-stressed brains of EOfAD mutation carriers than in non-carriers, leading to an earlier onset of Alzheimer’s disease. The idea of a “discontinuous cellular change of state” (Herrup, 2010) into overt Alzheimer’s disease (described here as a “stress threshold change of state”) has also been suggested by others (discussed in Herrup, 2010). We summarize this idea in **Figure 5**.

An appealing aspect of the idea of iron dysregulation contributing to the stress that ultimately triggers a transition into Alzheimer’s disease is that it might explain the minimal overlap between the EOfAD mutant loci and the LOsAD risk loci. This lack of overlap has led some to question whether EOfAD and LOsAD represent the same disease (Jayne et al., 2016) although both forms of Alzheimer’s disease show similar disease progression once symptoms become overt (Masters et al., 2015). We see that the EOfAD mutations might initially create metabolic stress by disrupting iron homeostasis leaving EOfAD brains with less capacity to adjust to the rising hypoxia caused by age-related vascular changes. The LOsAD risk loci broadly appear to affect autophagy/endolysomal function or inflammation and so either contribute to a milder form of iron “dyshomeostasis” (and reduce cells’ ability to cope with the effects of energy deficiency such as protein aggregation) or reduce the threshold at which inflammation becomes chronic and self-destructive (Richards et al., 2016). Possibly, the change of state into hypometabolism in brains with profound Alzheimer’s disease is a last-ditch attempt by cells to avoid death in the face of critically high oxidative stress levels.

Notably, a metabolic inversion similar to the Alzheimer’s disease stress threshold change of state model above has been described for the monogenetic neurodegenerative condition, Huntington’s disease, in a review by Browne and Beal (2004). This hypothesis proposes that early increases in glucose utilization observed in presymptomatic mouse models and in some human studies, may be a widespread cellular response to perturbation of normal Huntingtin protein function, while decreased glucose utilization observed in the striatum in later stages of the disease may be due to specific cellular dysfunction (Browne and Beal, 2004). There is a long history of observations of cellular iron dysregulation when the *HUNTINGTIN* (*HTT*) gene is mutated (Muller and Leavitt, 2014), and wild-type huntingtin is required for normal distribution of iron, and/or expression of iron-related gene products, in the embryos of mice (Dragatsis et al., 1998) and zebrafish (Lumsden et al., 2007), in adult mouse brain (Dietrich et al., 2017) and in mouse embryonic stem cells (Hilditch-Maguire et al., 2000; Jacobsen et al., 2011).

## App – An Expression Conundrum

An encouraging aspect of refocusing EOfAD analysis around changes in iron homeostasis is that it requires us to give greater attention to APP’s role in stress responses. Hypoxia causes oxidative stress that upregulates expression of APP, BACE1, and the PSENs (Sun et al., 2006; Wang et al., 2006; Zhang et al., 2007; Guglielmotto et al., 2009; Moussavi Nik et al., 2012, 2015; Villa et al., 2014) and can, thereby, greatly increase production of Aβ that can function as an antioxidant (Kontush, 2001; Kontush et al., 2001; Smith et al., 2002; Nadal et al., 2008; Baruch-Suchodolsky and Fischer, 2009) [although accumulated Aβ possibly generates ROS in the presence of Fe^2+^ via the Fenton reaction (Sutherland et al., 2013; Cheignon et al., 2017)]. Accumulating evidence also supports a role for Aβ as an antimicrobial peptide (Soscia et al., 2010; Kumar et al., 2016a,b). Aβ was reported to suppress the growth of *Candida albicans* and a variety of bacterial species at concentrations equal to, or lower than, another characterized human antimicrobial peptide, LL37, and depletion of Aβ from Alzheimer’s disease brain homogenates reduces the latter’s ability to inhibit growth of *C. albicans* (Soscia et al., 2010). An antimicrobial function for Aβ would explain its high structural conservation in most species of the tetrapod evolutionary lineage (Sharman et al., 2013; Moore et al., 2014).

That Aβ exhibits antioxidant and antimicrobial characteristics is consistent with the fact that inflammatory processes produce oxidative stress as a means of killing pathogens (Mittal et al., 2014). However, this also presents a conundrum: as mentioned previously, a rapid cellular and systemic (acute phase) response to bacterial infection is to sequester iron into cells and away from invading bacteria so as to inhibit their metabolism and growth (see, Urrutia et al., 2013 and reviewed in Soares and Weiss, 2015; Gozzelino and Arosio, 2016). While this explains why IL1 drives increased translation of the FERRITIN proteins to store iron as inactive Fe^3+^ (as described earlier), oxidative stress also increases APP transcription while IL1 increases APP translation. Increased full-length APP might be expected to stabilize FPN1 and promote iron export from, specifically, neurons in opposition to the action of HAMP (that drives FPN1 internalization and degradation (Nemeth et al., 2004; Ding et al., 2011)). Could it be that expulsion of “free” cytosolic iron from neurons via increased expression of APP promotes brain survival in the face of infection by allowing absorption of the iron by adjacent non-neuronal cells better equipped to sequester it? Simultaneously, increased cleavage of APP by β-secretase (encoded by *BACE*) may be important for diverting some of this increased APP protein into Aβ production for this peptide’s antioxidant and iron-sequestering capacity (**Figure 1**). Or does the increased β-secretase cleavage of APP due to inflammatory stress overwhelm the increase in APP transcription and translation to reduce levels of full-length APP and reduce iron export from neurons? Clearly, we need a much more detailed understanding of the relationship between iron homeostasis and the production of APP and Aβ at the cellular level during inflammatory responses in the brain.

A potential weakness in the idea of iron-dysregulation as unifying the effects of EOfAD mutations in *APP* and the *PSEN* genes, is that the various EOfAD mutations in APP are not all expected to produce the same effect on iron homeostasis. Some EOfAD mutations in APP (e.g., duplications and possibly those decreasing α-secretase cleavage) are expected to produce neuronal Fe^2+^ deficiency while others (e.g., those increasing β-secretase cleavage) might produce Fe^2+^ overload by decreasing normal APP function. However, the same criticism can be leveled at the Amyloid Hypothesis where the effects of γ-secretase site mutations are explained as causing changes in Aβ species length distribution while no explanation is clear for how *APP* duplication or mutations affecting the frequency of cleavage at the β-site can cause this. However, Arimon et al. (2015) showed that changes in Aβ length occur due to the effects of oxidative stress on the functioning of γ-secretase, while both cytosolic Fe^2+^ deficiency and overload would be expected to cause increased oxidative stress through interference with normal mitochondrial function. Thus, iron-dysregulation-driven oxidative stress potentially explains the common observation of changes in Aβ species length where the Amyloid Hypothesis cannot. We must note that most, if not all, of the EOfAD mutations in APP would appear to have the effect-in-common of increasing expression of APP’s transmembrane C99 fragment (that results from cleavage only at APP’s β-site) and Area-Gomez et al. (2018) have suggested that this may be critical for the increased MAM formation that is the basis of the MAM Hypothesis of Alzheimer’s disease (Schon and Area-Gomez, 2012). Interestingly, forced expression of C99 has metabolic effects since it appears to suppress ATP synthesis by oxidative phosphorylation in mitochondria while inhibition of C99 formation using an inhibitor of β-secretase increases oxidative phosphorylation (Pera et al., 2017).

## Concluding Remarks

The concept described above for unifying EOfAD mutation function should, of course, only be regarded as an outline requiring a great deal of enhancement and testing. For example, the brain consists of many different cell types with specialized roles and greatly differing energy metabolisms and how APP and the PSENs act within these cells types may differ considerably. EOfAD mutations of the *PSEN*s may affect metabolism and oxygen supply by a variety of independent routes such as direct effects on cells of the brain vasculature (Nakajima et al., 2006; Toussay et al., 2017) or via communication between neurons and brain microvasculature using unknown means (Gama Sosa et al., 2010). However, a conceptual focus on iron homeostasis coordinates many important aspects of Alzheimer’s disease including changes in metabolism/mitochondria, vascular function/blood flow/hypoxia, autophagy and inflammation and is not dependent on a central role for γ-secretase activity. We suggest that a concentration of effort in this area will give us valuable insights into the pathogenesis of Alzheimer’s disease.

## Author Contributions

This project was initiated by ML and all co-authors contributed ideas, contributed text, and edited this work. Illustrations were drafted by ML and then adjusted after input from the co-authors.

## Conflict of Interest Statement

The authors declare that the research was conducted in the absence of any commercial or financial relationships that could be construed as a potential conflict of interest.
